# Multiple Vaccinations and the Enigma of Vaccine Injury

**DOI:** 10.3390/vaccines8040676

**Published:** 2020-11-12

**Authors:** Anthony R. Mawson, Ashley M. Croft

**Affiliations:** 1 Department of Epidemiology and Biostatistics, School of Public Health, College of Health Sciences, Jackson State University, Jackson, MS 39213, USA; 2 School of Pharmacy and Biomedical Sciences, University of Portsmouth, White Swan Road, Portsmouth PO1 2DT, UK; ashleycroft@doctors.org.uk

**Keywords:** vaccination, injury, liver, retinoids, metabolism, disease, allergy, neurodevelopmental disorders, infections, outcomes, Gulf War illness

## Abstract

A growing number of vaccines are administered at the same time or in close succession, increasing the complexity of assessing vaccine safety. Individual vaccines are assumed to have no other effect than protection against the targeted pathogen, but vaccines also have nonspecific and interactive effects, the outcomes of which can be beneficial or harmful. To date, no controlled trials and very few observational studies have determined the impact of vaccination schedules on overall health. The balance of the risks and benefits from mass vaccination therefore remains uncertain. Recent studies worryingly suggest links between multiple vaccinations and increased risks of diverse multisystem health problems, including allergies, infections, and neuropsychiatric or neurodevelopmental disorders. Here, we propose that, in susceptible persons, multiple vaccinations activate the retinoid cascade and trigger apoptotic hepatitis, leading to cholestatic liver dysfunction, in which stored vitamin A compounds (retinyl esters and retinoic acid) enter the circulation in toxic concentrations; this induces endogenous forms of hypervitaminosis A, with the severity of adverse outcomes being directly proportional to the concentration of circulating retinoids. In very low concentrations, vitamin A and its major metabolite retinoic acid contribute to immune function and to the process of immunization, whereas excess vitamin A increases the risk of adverse events, including common “side-effects” as well as chronic adverse outcomes. The increasing rates of allergy, ear infections, and neurodevelopmental disorders (NDDs) in countries with high rates of vaccination could be related to mass vaccination and to its impact on liver function and vitamin A metabolism, collectively representing endogenous manifestations of hypervitaminosis A. Further studies of health outcomes in vaccinated and unvaccinated groups are urgently needed, to increase understanding of the pathophysiology and treatment of vaccine injury, to identify the risk factors and screen for vaccine injury, to inform public health policy on potential hazards related to vaccination schedules, and to optimize the safety and benefits of vaccines.

## 1. Benefits and Current Status of Vaccines

Vaccines given to infants and young children in the first two decades of the 20th century are estimated to have prevented 322 million illnesses, 21 million hospitalizations, and 732,000 deaths during their lifetimes [1]. The discovery in 1962 of the first human cell strain used to produce licensed human virus vaccines (WI-38) is also credited with saving 10.3 million lives [2]. The primary benefit of vaccination—disease prevention—is considered one of the greatest public health achievements of the 20th century [3]. Vaccines for preventing other global scourges such as HIV, tuberculosis, and malaria, and for the current pandemic of COVID-19, remain high priorities for development [4,5]. 

In recent decades, as new vaccines have been developed, the childhood immunization schedule has been expanded and accelerated. As a result, multiple vaccinations are administered during a single office visit or in close succession. Like all medicines, vaccines can have adverse effects, but life-threatening events are considered so rare that the full schedule of vaccinations is required for children to attend daycare and schools [6]. 

The currently recommended USA schedule involves 69 doses of 16 vaccines starting on the day of birth with the first dose of the hepatitis B vaccine and continuing up to age 18, with 50 doses given before age 6 [7]. This represents a three-fold increase in vaccines compared to the schedule in 1983 [8]. To reduce the number of required injections, many combination vaccines have been developed [9].

Vaccines are presumed to have no other effect than protection against the targeted pathogen, aside from rare adverse effects. Studies have not been performed to evaluate the overall impact of the immunization schedule itself. In fact, no difference in health outcomes would be expected between vaccinated and unvaccinated groups, except for reduced rates of the targeted infectious diseases. However, individual vaccines have nonspecific and interactive effects on health, which can be either positive or negative, in that certain vaccines have beneficial nonspecific effects, whereas others increase morbidity and mortality. For instance, the standard measles vaccine and the Bacillus Calmette-Guerin BCG, smallpox, and polio vaccines have unintended beneficial effects, preventing other infectious and noninfectious diseases, whereas other vaccines, such as the diphtheria–tetanus–pertussis (DTP) vaccine, increase mortality from unrelated diseases. Alarmingly, it was recently concluded that “All currently available evidence suggests that DTP vaccine may kill more children from other causes than it saves from diphtheria, tetanus or pertussis” [10]. The different nonspecific outcomes depend partly on the order of vaccine administration, on whether the vaccines are live or inactivated, and on concomitant supplementation with vitamin A [11]. The existence of these nonspecific and interactive effects challenges current understanding of the mechanism of action of vaccines and how they affect the immune system [12,13].

Since 1986, >USD 4.3 billion has been awarded in compensation for vaccine injury by the National Vaccine Injury Compensation Program, but these reports are believed to represent <1% of serious adverse events [14], suggesting that such events are much more common than are officially recorded. Awards for compensation do not imply that vaccines caused the alleged injuries. Most awards result from a negotiated settlement between the parties in which it is concluded that the outcome and the circumstances match those of previous cases, as set forth in the Vaccine Injury Table [15]. The data for such cases derive from reports to the Vaccine Adverse Effects Reporting System (VAERS), filed voluntarily by physicians or parents. The Table includes a list of vaccines and the “injuries, disabilities, illnesses, conditions, and deaths” claimed to have resulted from the administration of such vaccines. For deciding eligibility for compensation under the Program, the Table also defines the allowed periods of time during which the first symptoms or manifestations had to have occurred after vaccine administration. For instance, the Table includes encephalopathy as a vaccine injury, defining it as a change in mental or neurologic status first manifested during the applicable time period and that persists for at least 6 months from the date of vaccination. Another tracking system for vaccine adverse effects, the Vaccine Safety Datalink (VSD), is a collaboration between the CDC and nine healthcare organizations [16]. As vaccinations are a covered benefit of the VSD health plans, and vaccine coverage rates are higher than national coverage estimates, this highly vaccinated population has limited numbers of unvaccinated children for comparison purposes [17].

## 2. Growing Safety Concerns

Little scientific evidence exists upon which to base claims for compensation. Numerous epidemiological studies have found no association between the few selected vaccines that have been studied (most notably, the combined measles, mumps, and rubella vaccine) and neurodevelopmental disorders [18,19,20]. The long-term effects and safety of vaccine ingredients such as adjuvants and preservatives, particularly aluminum-based adjuvants, are also being increasingly questioned [21,22]. Likewise, little is known about the safety of vaccines against diseases that can be lethal for individuals but have a lesser impact on population health, such as the group B meningococcus vaccine [23]. Research on understanding the biological mechanisms underlying vaccine injury remains limited, and there is no accepted mechanism by which vaccines can induce disorders such as autism [24].

Stakeholders have expressed concerns that “infants get more vaccines than are good for them”. However, the number of antigens (i.e., inactivated or dead viruses and bacteria, or altered bacterial toxins) received by children in the USA and Europe has declined compared to those in immunization programs during the 1960s or 1980s [25]. Today, the USA schedule comprises 11 antigens, and those in some European countries comprise nine on average. This decline in the number of antigens is shown in Table 1.

Although the number of antigens delivered in vaccination schedules has declined, vaccinations are commonly given at younger ages, or as multiple vaccines administered concurrently. As vaccines have become more complex, the non-antigen components in vaccines have increased both in number and complexity, such as the adjuvant aluminum, which is designed to increase immunogenicity.

Other concerns of stakeholders related to vaccines include the absence of studies on the health outcomes of fully vaccinated versus partially vaccinated and unvaccinated children; the paucity of studies on the immunization schedule itself; the lack of information on the effects of multiple vaccinations given at a single visit [26]; and whether vaccines could be contributing to the unexplained increases in the rates of allergy, asthma, and neurodevelopmental disorders (NDDs) [27,28,29,30]. The balance of the risks and benefits from the receipt of the recommended vaccination schedule therefore remains uncertain.

In the face of this uncertainty, the first author and colleagues studied vaccinated and unvaccinated homeschooled children, among whom vaccination coverage rates are lower than in the general population [31,32]. The aims were to compare health outcomes and exposures to determine if any association found between vaccination and NDDs remained significant after controlling for other significant factors. The survey targeted homeschooling mothers in the states of Florida, Louisiana, Mississippi, and Oregon. The mothers were asked to complete an anonymous questionnaire on their 6- to 12-year-old biological children regarding pregnancy-related exposures, birth history, vaccinations, physician-diagnosed illnesses, medications, and the use of health services. NDDs were defined as having one or more of the following: A physician diagnosis of autism spectrum disorder (ASD), attention deficit hyperactivity disorder (ADHD), or a learning disability. A sample of 666 children was obtained, of which 261 (39%) were unvaccinated. The vaccinated were significantly less likely than the unvaccinated to have been diagnosed with chickenpox and pertussis; they were, however, significantly more likely to have been diagnosed with:

1. Allergic rhinitis (Odds Ratio: 30.1; 95% Confidence Interval: 4.1, 219.3);
2. Eczema (OR: 2.9; 95% CI: 1.4, 6.1);
3. A middle ear infection (OR: 3.8; 95% CI: 2.1, 6.6);
4. Pneumonia (OR: 5.9; 95% CI: 1.8, 19.7);
5. An NDD (OR: 3.7; 95% CI: 1.7, 7.9).

The vaccinated were also more likely to have used allergy medication (20% vs. 1.2%, *p* < 0.001; OR: 21.5; 95% CI: 6.7, 68.9), been fitted with ventilation ear tubes (3.0% vs. 0.4%, *p* = 0.018; OR: 8.0; 95% CI: 1.0, 66.1), and spent one or more nights in a hospital (19.8% vs. 12.3%, *p* = 0.012; OR: 1.8; 95% CI: 1.1, 2.7). Between fully vaccinated and unvaccinated children, partially vaccinated children had intermediate odds of diagnosis with allergic rhinitis, eczema, and NDD, suggesting a dose–response relationship between vaccinations and these adverse effects.

The factors associated with NDDs in unadjusted analyses were vaccination (OR: 3.7; 95% CI: 1.7, 7.9); male gender (OR: 2.1; 95% CI: 1.1, 3.8); living within 1–2 miles of a furniture manufacturing factory, hazardous waste site, or lumber processing factory (OR: 2.9; 95% CI: 1.1, 7.4); maternal use of antibiotics during pregnancy (OR: 2.3; 95% CI: 1.1, 4.8); and preterm birth (OR: 4.9; 95% CI: 2.4, 10.3). After adjustment, the factors that remained significantly associated with NDDs were vaccination (OR: 3.1; 95% CI: 1.4, 6.8), male gender (OR: 2.3; 95% CI: 1.2, 4.3), and preterm birth (OR: 5.0; 95% CI: 2.3, 11.1). In a final adjusted model, controlling for the interaction of preterm birth and vaccination, the factors that remained significantly associated with NDDs were vaccination, nonwhite race, and male gender. Preterm birth was not significantly associated with NDDs in the absence of vaccination. However, preterm birth combined with vaccination was associated with a synergistically 6.6-fold increased odds of NDDs (95% CI: 2.8, 15.5).

Other studies supporting an association between multiple vaccinations and adverse health outcomes in children are summarized below:

1. In a study based on 38,801 reports to VAERS, a linear relationship was observed between the number of vaccine doses administered at one time and the rate of hospitalization and death in infants after receiving vaccinations. The hospitalization rate for vaccine injury increased from 11% for two doses to 23.5% for eight doses (r^2^ = 0.91). The case-fatality rate increased significantly from 3.6% for 1–4 doses to 5.4% for those receiving 5–8 doses [33].
2. The USA has the highest Infant Mortality Rate (IMR) among 33 other nations; it also has the highest number of required doses of vaccines for infants among these nations. An examination of the immunization schedules of these 34 countries showed a strong correlation between IMRs and the number of routinely administered doses of vaccines (r^2^ = 0.70, *p* < 0.0001) [34].
3. In a study comparing health outcomes among vaccinated and unvaccinated US children born between 2005 and 2015, based on medical records from three medical practices, vaccination before age 1 was significantly associated with developmental delays (OR: 2.18; 95% CI: 1.47–3.24), asthma (OR: 4.49), and ear infections (OR: 2.13) [35].
4. A study examined the association between the proportion of children receiving the recommended vaccines by age 2 in each state and the prevalence of autism or speech disorders [36]. After controlling for family income and ethnicity, the proportion of children receiving the recommended vaccinations was directly related to the prevalence of autism and of speech disorders. A 1% increase in the vaccination rate in each state was associated with an additional 680 children having either outcome.

An ill-defined pattern of multisystem adverse outcomes, known as Gulf War illness (GWI), is seen in veterans of the 1990–1991 Persian Gulf War. Over 25% of veterans, among them both deployed and nondeployed veterans, continue to experience diverse signs and symptoms that include allergies, frequent infections, cognitive disorders (including memory impairment), chronic musculoskeletal pain, fatigue, muscle weakness, headache, anxiety and depression as well as respiratory, gastrointestinal, dermatologic and reproductive problems [37]. Potential risk factors included exposure to several neurotoxins and the anticholinergic drug pyridostigmine bromide (PB), which was routinely taken as prophylaxis against the nerve agent soman. No association was found between deployment to the war zone and specific health effects, or between specific exposures during deployment and health effects related to GWI. This was true whether the veterans were from the US, the UK, Canada, Australia, or Denmark [38]. “Stress” and related psychiatric etiologies were ruled out [39]. Veterans of the 1990–1991 Gulf War also appear to be at increased risk of chronic illness in later life, particularly from high blood pressure, high cholesterol, heart attacks, diabetes, strokes, arthritis, and chronic bronchitis [40]. 

A review of the literature on GWI [41] noted that nearly all Gulf War veterans reported getting at least one vaccine for deployment, 70% received over five vaccines, and 30% received over 10 vaccines [42]. Several studies have linked GWI to the receipt of multiple vaccines:

1. A survey of over 4000 British Gulf War male veterans reported that “vaccination against biological warfare and multiple routine vaccinations were associated with GWI in the Gulf War cohort.” [43].
2. Among 1548 Kansas GW veterans and controls (non-GW), the rate of GWI among vaccinated GW veterans was 11 times higher than among non-GW veterans who did not receive vaccines. Vaccinated non-GW veterans had significantly higher rates of chronic pain, neurological, and gastrointestinal problems, and nearly four-fold higher odds of GWI than non-GW veterans who did not receive vaccines. It was concluded that vaccines used during the war may be a contributing factor to the excess morbidity among Gulf War veterans [44].
3. A follow-up, medical record-based study of British GW veterans 6–8 years after deployment reported that receipt of multiple vaccinations was associated with five-fold increased odds of GWI [45].
4. A study of nearly 8000 personnel with the UK forces in the Gulf War reported that the number of vaccinations received was directly associated with higher scores for skin and musculoskeletal complaints [46].
5. A study of outcomes among 1456 Australian GW veterans and a control group of veterans who were not deployed (*n* = 1588) found that the number of symptoms reported in the past month by GW veterans was associated with vaccinations in a dose-dependent manner. Those with the highest number of symptoms had at least 10 vaccinations [47].

In summary, studies to date collectively suggest that vaccinated children, although less susceptible to certain vaccine-preventable infections than unvaccinated children, as expected, are significantly more likely to be diagnosed with chronic conditions; these collectively include allergic rhinitis, eczema, asthma, middle ear infections, pneumonia, developmental delays, and neurodevelopmental disorders (ASD, ADHD, and learning disabilities, including speech-and-language disorders). Secondly, exposure to full versus partial vaccination is associated with higher rates of these conditions and with an increased risk of dying in infancy. Thirdly, the receipt of multiple vaccinations prior to deployment has been found to be associated with Gulf War illness. In-theater exposures may account for any single individual veteran’s ill health, but many veterans of the same era who were not deployed overseas continue to suffer the same or similar symptoms. The features of GWI also overlap with those of chronic fatigue and multiple chemical sensitivity, in all of which liver dysfunction has been implicated [48]. Veterans with GWI are reported to have increased liver enzymes and increased clinical diagnoses of liver disease, including a 7.5-fold increased odds of obstructive liver disease compared to veterans without multisystem illness [49].

## 3. Unifying Hypothesis on the Pathogenesis of Vaccine Injury

Clues to the pathogenesis of vaccine injury include the fact that > 80% of vitamin A (vA) is stored in the liver in the form of retinyl esters, and these vA compounds (collectively termed retinoids) can be extremely toxic to cell membranes if they are released from the liver unbound to protein following disease or injury [50]. Apoptosis has been suggested as the underlying pathophysiological mechanism of the adverse effects of vA poisoning, including birth defects [51]. These observations led us to propose that exposure to multiple vaccinations, possibly with additional chemical insults of a liver-damaging nature, plausibly explains GWI and its observed chronicity [52]. Based on this hypothesis, the suggested pathogenesis is a chemically induced impaired liver function involving activation of the retinoid cascade in the liver. This inhibits the secretion of retinol-binding protein 4 (RBP4), thereby lowering serum retinol concentrations, but simultaneously increases the accumulation and expression of retinoids in the liver, leading to apoptotic hepatocellular damage and the entry of retinyl esters and retinoic acid into the circulation in toxic concentrations. The result is an endogenous chronic form of hypervitaminosis A. 

The diverse features of GWI include neuropsychiatric and gastrointestinal signs and symptoms, pain, headaches/migraines, abnormal fatigue, respiratory and bone problems such as osteoporosis and fractures, and reproductive problems such as higher rates of miscarriage and significant excesses of birth defects, spontaneous abortion, and stillbirth. These features closely mirror those of acute and chronic hypervitaminosis A. GWI thus represents a virtually paradigmatic case of multiple vaccine- and liver damage-induced hypervitaminosis A.

Here, we extend the hypothesis to propose that acute vaccine injury and the chronic adverse outcomes from individual and particularly multiple vaccines administered at the same time or in close succession are likewise due to liver damage and resulting hypervitaminosis A. Unlike GWI, which has been the subject of many focused investigations, little is currently known about the spectrum of signs, symptoms, and diagnoses relating to vaccine injury. The limited evidence to date suggests that vaccinated children may be at risk for multisystem illnesses like those of veterans with GWI (allergies, infections, and neurodevelopmental/neuropsychiatric disorders). New studies are therefore awaited on liver function, vA metabolism, and retinoid concentration profiles both in veterans with GWI and in children with conditions that are thought to be vaccine-related. 

**Figure 1:**
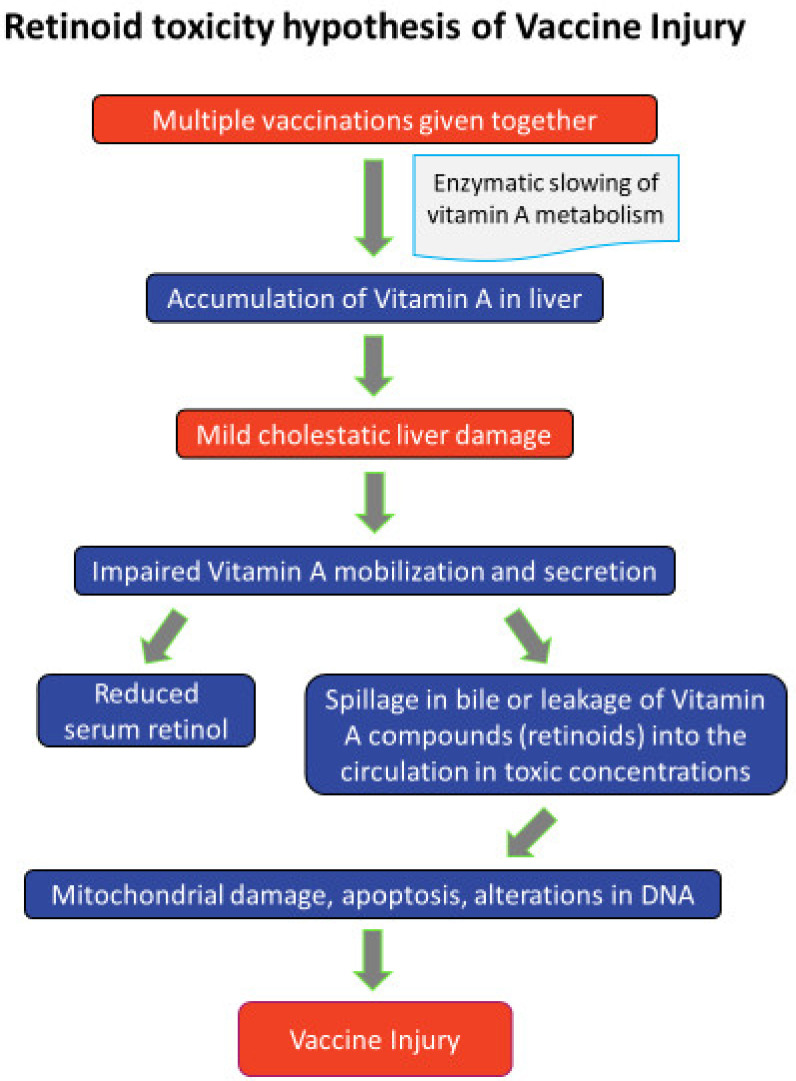
Retinoid toxicity hypothesis of vaccine injury.

The retinoid toxicity hypothesis of vaccine injury is depicted in the figure below (Figure 1).

*Allergy*—A nationwide case-control study of Japanese individuals 20–65 years of age with physician-diagnosed multiple chemical sensitivity (MCS) showed that MCS was associated with ≥ 11 vaccinations in the past 10 years, caesarean section delivery, and multiple moves to new homes [52]. In a mouse model of allergic dermatitis, the expression of Interleukin-4 (IL-4) was increased in inflamed skin, demonstrating an altered immune response. Gene expression analysis showed alterations in cutaneous retinoid metabolism and retinoid-mediated signaling. Retinoic acid levels and synthesis were increased, as shown by the elevated expression of retinaldehyde dehydrogenases [53]. These findings suggest that increased Retinoic Acid Receptor- (RAR)-mediated signaling in allergen-induced dermatitis may contribute to the development and/or maintenance of allergic skin diseases. As noted in our review on GWI [41], mast cells are increased in patients with atopic dermatitis and express high levels of retinoic acid receptor-alpha. Retinoic acid (RA) also interferes with the proliferation of skin mast cells and promotes their degranulation, supporting the concept that RA has a pro-allergic and pro-inflammatory-maintaining function in skin mast cells. 

*Neuropsychiatric disorders*—A study of insurance claims data on privately insured US children showed that vaccinations are associated with obsessive–compulsive disorder (OCD), anorexia nervosa (AN), anxiety disorder, chronic tic disorder, attention deficit hyperactivity disorder (ADHD), major depressive disorder, and bipolar disorder. Those with newly diagnosed AN were more likely than matched controls to have had any vaccination in the previous 3 months (hazard ratio (HR): 1.80; 95% CI: 1.21–2.68), and those with AN, OCD, and anxiety disorder were more likely than controls to have received influenza vaccinations during the previous 3, 6, and 12 months. HRs >1.40 were also found for hepatitis A vaccination with OCD and AN, for hepatitis B with AN, and meningitis with AN and chronic tic disorder [54]. Other research has found that the therapeutic use of 13-cis-RA (isotretinoin) is associated with the onset of depression, psychosis, and suicide [55], and both depression and cognitive disturbances in 1% to 11% of patients with acne [56]. The neuropsychiatric symptoms of acute vA poisoning include drowsiness, irritability, severe headaches, nausea, and various forms of impulsive and irrational behavior [57]. 

*Neurodevelopmental disorders*—A review of the role of retinoids in NDDs [58] includes the suggestion that an abnormality in the interplay between retinoic acid and sex hormones may cause ASD [59], and that RA and related signaling pathways could provide a target for the treatment of ASD. Studies are awaited on vA concentration profiles in children with NDDs.

*Gastrointestinal*—There is suggestive evidence of associations between the poliomyelitis vaccine and risk of Crohn’s disease (Relative Risk, RR: 2.28; 95% CI, 1.12–4.63) and of ulcerative colitis (RR: 3.48; 95% CI, 1.2–9.71) [60]. Chronic digestive symptoms, along with neuropsychological dysfunction, were reported by veterans returning from the 1990–1991 Gulf War [61]. Retinoids are linked to a wide range of adverse and often severe gastrointestinal effects [62].

*Pain/headache/migraine*—Headaches are a common side-effect of many vaccines. A study of the VAERS database showed that headaches were the fifth most frequently reported symptom after fevers, injection site reactions, pain, and rashes. Of 28,286 reports of headaches, 29% also included nausea or vomiting. The onset was within one day of vaccination in 69% of cases [63]. Severe headaches (pseudotumor cerebri) and skeletal pain are common manifestations of hypervitaminosis A [64].

*Fatigue and respiratory problems*—Chronic fatigue syndrome includes disabling fatigue, headaches, concentration difficulties, and memory deficits and can occur following vaccinations, including the Measles-Mumps-Rubella (MMR), Pneumovax, influenza, hepatitis B virus (HBV), tetanus, typhoid, and poliovirus vaccines [65]. DTaP vaccination is linked to soreness or swelling at the site of injection, with a fever, feeling tired, a loss of appetite, and occasional vomiting; more serious reactions include seizures, continuous crying or a high fever (over 105 °F), limb swelling, and, very rarely, long-term seizures, coma, encephalopathy, permanent brain damage, severe hypersensitivity reactions, or death [66]. Symptoms of severe fatigue and other signs of chronic hypervitaminosis A can appear 2–3 months and even years after excessive vA exposure through dietary intake, supplementation, or endogenous sources [67]. Treatment with synthetic retinoids can also cause fever and general fatigue, dyspnea, and respiratory distress, in some cases requiring endotracheal intubation and mechanical ventilation [68].

## 4. Vitamin A and Immune Function

Vitamin A and its major metabolite retinoic acid (RA) are critically involved in immune function [69,70]. Appropriate immune responses depend on dendritic cells synthesizing RA from gut-associated lymphoid tissues. RA is critical for generating immunoglobulin A-secreting B cells, the development of immune tolerance, and Foxp3 regulatory T lymphocyte (Treg) cells [71]. RA also plays a role in the differentiation and survival of Th17 cells, which produce IL-17, IL-21, and 1L-22 and enhance bacterial and fungal infection control [72] as well as T cell-dependent antibody responses [73]. Low RA promotes T-helper (Th1) cells, whereas higher concentrations promote Th2 cells [74]. vA and RA are also important in immunization, which involves collaboration between antigen-presenting cells, T cells, and B cells [70,75]. 

Retinoids generally reduce T cell proliferation but increase B cell proliferation; hence, low vitamin A stores result in a poor response to immunization, with generally reduced antibody responses to immunization with T cell-dependent antigens. The impaired response to many vaccines and the need for vaccine adjuvants to increase immunogenicity in the neonate [76,77] is mainly attributed to maternal antibodies. It could also be explained by the fact that vA concentrations in the liver are low in infancy, especially in the first 6 months of life [78]. 

RA affects many B cell processes and interacts with co-stimulatory signals such as cytokines and adjuvants in ways that can be additive or synergistic [79]. In mice, the antibody titers produced after immunization with tetanus toxoid (TT), a known T cell-dependent antigen, were increased when RA was administered orally at the time of first immunization, and increased even further after the second immunization, several weeks later [80]. These observations are relevant to understanding the role of vA in the humoral antibody response to immunization, the hallmark of successful vaccination, and in understanding adverse responses to vaccines. Oral vA supplementation significantly enhances anti-TT antibody responses, with a greater effect when combined with RA, suggesting that vA augments immune responses [81]. Indeed, RA could serve as an effective mucosal adjuvant in vaccines [82] and, under conditions of sufficiency or excess, can be pro-inflammatory and potentially harmful. For instance, in an experimental model of gluten-related enteropathy (a mouse model of celiac disease), RA produced inflammatory cytokines that reduced the capacity of mucosal dendritic cells to promote Treg cell production and led to exaggerated responses to dietary glutens [83]. Reports also link pharmacologic retinoid treatment to severe intestinal inflammation [84]. 

### 4.1. Vaccination and Disease Pathogenesis 

Vaccination may reproduce, to a normally minor extent, the pathogenesis of many infectious diseases, such as flu-like signs and symptoms. Based on our hypothesis, this involves a mild degree of activation of retinoid pathways and generally mild acute “side-effects” that are manifestations of hypervitaminosis A; injury due to vaccines involves retinoid overaction and/or accumulation, liver damage, and the spillage of higher amounts of stored retinoids into the circulation. Just as in the case of natural infection by a single agent, the mobilization and secretion of RBP from the liver are impaired, leading to lowered serum retinol concentrations. Multiple vaccinations administered at the same time or in close succession—as in the childhood vaccination schedule—could therefore induce greater liver dysfunction and retinoid accumulation than one or very few vaccinations, resulting in adverse effects that reproduce the spectrum of acute features associated with infectious diseases and their complications, including tissue damage and hypersensitivity disorders. This could explain why vaccine injury and associated illness (and the interpretation of GWI in particular) have been sources of diagnostic uncertainty. 

Evidence of liver involvement in vaccine injury remains limited and awaits further study. Adjuvanted (aluminum hydroxide) hepatitis B vaccine exposure in mice was reported to upregulate key genes encoding apoptosis-inducing caspases, suggesting that cell death occurs via the loss of mitochondrial integrity [85]. The hepatitis B vaccine also altered the expression of 144 genes in the mouse liver within 1 day of vaccination, some of which were related to inflammation and metabolism [86]. Among children receiving the hepatitis B vaccination, according to findings from the 1993 National Health Interview Survey, the age-adjusted odds ratio for liver problems was 2.35 compared with unvaccinated controls [87]. 

### 4.2. Susceptibility to Vaccine Injury 

Our model suggests that susceptibility to vaccine injury is increased in persons with high background stores of vA in the liver; that is, such stores may increase the risk of tissue damage via activation of the retinoid cascade within the liver. Excess vitamin A can cause liver damage due to apoptosis [88], as shown by the loss of mitochondrial membrane potential and the activation of caspases. Aluminum salts may also increase RA concentrations and expression, since some of their effects, e.g., dendritic cell activation and the stimulation of uric acid production, are those of RA itself [89]. Based on this model, retinoid-induced liver damage related to multiple vaccinations leads to a transient form of cholestatic liver dysfunction in which stored retinoids enter the circulation, causing a broad range of adverse effects as manifestations of vA toxicity. Bile normally drains from the liver through the gall bladder and common bile duct into the duodenum. However, when regurgitated into the circulation, bile may be particularly hazardous, as it contains four-fold higher concentrations of retinol than serum and other extrahepatic tissues [90]. 

Our hypothesis is that vaccination-associated adverse effects in young children are due to liver damage and vitamin A intoxication. For instance, vomiting and bulging fontanelles due to increased intracranial pressure are symptoms of hypervitaminosis A [91] and are often reported following vaccination [92,93,94]. Features of autism spectrum disorder (ASD) also resemble those of acute and chronic hypervitaminosis A. These features of ASD include the following:

1. Gastro-intestinal inflammation [95,96].
2. Heightened sensitivity to sounds [97].
3. Altered food preferences, sensitivities/allergies, self-stimulatory or self-injurious behavior, and seizures [98].
4. Mitochondrial dysfunction [99,100].
5. Bone changes, including reduced bone mineral density and bone cortical thickness in boys, as well as increased risks of hip, spine, and forearm fractures [101,102].
6. Abnormal posturing and walking on tiptoes [103], likely indicating idiopathic intracranial hypertension, which can be induced by vitamin A intoxication [104]. This can cause foot drop, which forces the individual to walk on tiptoes.

Acute or chronic vA intoxication has also been linked to fevers, seizures, lethargy, slowed growth or growth arrest, learning and speech disorders, impaired attention, emotional instability, depression, aggression, and self-injury [105,106,107]; mitochondrial dysfunction [108]; inflammatory bowel disease [84]; and bone disorders [109]. Susceptibility to vaccine injury and resulting chronic illnesses may be due in part to previous exposure to retinoids, including vA supplementation itself. For instance, children who received vitamin A with the diphtheria, tetanus, and pertussis (DTP) vaccine were at greater risk of death than children who received vA alone or received nothing [110].

## 5. Conclusions

Many parents are questioning the safety of vaccines, leading the World Health Organization (WHO) in 2019 to declare “vaccine hesitancy” as one of the 10 biggest threats to global health, potentially reversing the progress made in tackling vaccine-preventable diseases [111]. The WHO declaration assumes that vaccine schedules are sufficiently safe to be required for school and daycare attendance, and that vaccine-hesitant parents are either uninformed or misguided. However, the growing number of vaccines administered at the same time or close together has increased the complexity of assessing vaccine safety [112]. Vaccines have nonspecific and interactive effects, the outcomes of which can be either beneficial or harmful, and the balance of long-term risks and benefits from mass vaccination remains uncertain. Recent studies also worryingly suggest links between the receipt of multiple vaccinations and increased risks of diverse multisystem health problems in children and adults. In the present state of knowledge it would therefore be premature to suggest that mandatory vaccination would be “ethically justified”, as if it were analogous to ethically justified mandatory seat belt use in motor vehicles [113].

We have proposed that acute injury and chronic multisystem illness related to vaccines involve liver dysfunction and the release of stored vA compounds into the circulation in toxic concentrations, leading to endogenous forms of hypervitaminosis A. In very low concentrations, vA and its major metabolite retinoic acid contribute to organogenesis, immune function, and immunization, involving collaboration between antigen-presenting cells, T cells, and B cells. In excess, vA increases the risk of adverse events, including common “side-effects” as well as chronic adverse outcomes that may be related to the accumulation of retinoids in different tissues. Multiple vaccinations and their ingredients, notably aluminum-based adjuvants, administered together could therefore have a proportionally greater impact on vA metabolism and liver function than single vaccines, and thereby increase the risks of acute and chronic adverse effects. 

The increasing rates of allergy, ear infections, and NDDs [26,27,28,29,114,115,116] in countries with high rates of vaccination suggest that these epidemics could be related to mass vaccination and represent endogenous manifestations of hypervitaminosis A. Tests of the hypothesis could include case-control studies of children and adults with these conditions and are expected to show that “cases” with ostensibly different conditions have had greater exposure to vaccinations and show evidence of liver dysfunction and of vitamin A toxicity compared to controls. In a recent paper, we proposed a new hypothesis for understanding rubella virus infection, congenital rubella syndrome (CRS), and the link to autism [117]: that rubella infection occurring early in pregnancy, which is strongly associated with congenital rubella syndrome (CRS), is due to maternal liver dysfunction and exposure of the fetus to excess vA. It was further suggested that post-natal influences of a liver-damaging nature, possibly including multiple vaccinations, can induce CRS-like features as a function of vA toxicity, but without the associated dysmorphogenesis.

Further studies of health outcomes in vaccinated and unvaccinated groups are urgently needed: to increase understanding of the pathophysiology and treatment of vaccine injury; to identify the risk factors and to screen for vaccine injury; to inform public health policy on potential hazards related to vaccination schedules; and to optimize the safety and benefits of vaccines.

## Figures and Tables

**Table 1:** Number of immunogenic pathogens/polysaccharides in vaccines over the past 100 years.

		Number of Proteins		Protein and Polysaccharides
Vaccine	1900	1960s	1980s	2000s
Smallpox	−200	−200		
Diphtheria		1		
Tetanus		1	1	1
Whole-cell pertussis		−3000	−3000	
Acellular pertussis				2-5
Poliomyelitis		15	15	15
Measles			10	10
Mumps			9	9
*Haemophilus influenzae*				2
Hepatitis B				1
Varicella				69
Pneumococcus				8

Source: Aronson [25].

## Note

An early version of this paper was presented by the first author at a conference in Rome on “Vaccinating in Safety”, sponsored by the Italian National Order of Biologists, 15 January 25 2019.
