# Correction: Mawson, A.R.; Croft, A.M. Multiple Vaccinations and the Enigma of Vaccine Injury. *Vaccines* 2020, *8*, 676

**DOI:** 10.3390/vaccines14020163

**Published:** 2026-02-10

**Authors:** Anthony R. Mawson, Ashley M. Croft

**Affiliations:** 1 Department of Epidemiology and Biostatistics, School of Public Health, College of Health Sciences, Jackson State University, Jackson, MS 39213, USA; 2 School of Pharmacy and Biomedical Sciences, University of Portsmouth, White Swan Road, Portsmouth PO1 2DT, UK; ashleycroft@doctors.org.uk

The authors would like to make the following correction to the published paper [1].

When citing reference 44 in Section 2 “Growing Safety Concerns”, instead of quoting the reference as concluding that vaccines “were a contributing factor to the excess mortality among Gulf War veterans”, the statement should have read as follows: “vaccines used during the war may be a contributing factor” to the excess mortality among Gulf War veterans. Therefore, the sentence “It was concluded that vaccines “were a contributing factor to the excess morbidity among Gulf War veterans” [44]” should be corrected to “It was concluded that vaccines used during the war may be a contributing factor to the excess morbidity among Gulf War veterans [44]”.

The authors state that the scientific conclusions are unaffected. This correction was approved by the Academic Editor. The original publication has also been updated.

## References

[1] Mawson A.R., Croft A.M. (2020). Multiple Vaccinations and the Enigma of Vaccine Injury. Vaccines.

